# The importance of diverse multiomics datasets and analyses

**DOI:** 10.1172/JCI184350

**Published:** 2024-11-01

**Authors:** Laura J. Rasmussen-Torvik

The past 15 years have seen enormous technical advances resulting in researchers having the ability to efficiently and economically generate large-scale -omics data (genetic, methylation, and proteomic data, to name just a few) on hundreds or thousands of samples provided by volunteers in many different types of research studies. Simultaneously, efforts by the NIH and other funders have made this data rapidly available to researchers all over the world through expanding data repositories such as https://sharing.nih.gov/accessing-data/accessing-genomic-data/accessing-genomic-data-from-nih-repositories, https://www.proteomicsdb.org/ and https://ngdc.cncb.ac.cn/methbank These databases have enabled critical analyses exploring pathways of disease. However, some analyses arising from public databases are plagued with issues such as lack of generalizability and lack of replication. A particular challenge is that the easiest to use datasets (those with the lowest barriers to access or the best documentation for use) may not always include the best data to address a given hypothesis. The UK Biobank, for example, has facilitated large numbers of important discoveries, but there are limitations, such as, the ability to analyze outcomes that require information outside of hospital inpatient data (1). Furthermore, the relative paucity of genetic data generated in non-White populations in these databases has led to in far too few genetic analyses published in non-White populations (2), resulting in disparities that affect genetic risk prediction in non-White populations (3, 4) and, fundamentally, limiting discovery opportunities (5).

In this issue of the JCI, Tahir and colleagues (6) do not, by any means, take the easiest approach to large-scale protein quantitative loci (pQTL) and phenome-wide association study (PheWAS) analyses. Instead, they thoughtfully utilized data from two different cohorts (Jackson Heart Study and Multi-Ethnic Study of Atherosclerosis) and two different biobanks (All of Us and BioMe) to assemble an analytic dataset including large numbers of individuals with African ancestry. Using assembled cohort data, they identified cis-pQTLs (some among variants enriched in those with African ancestry) and then, using assembled biobank data, they performed a PheWAS and discovered important associations between identified cis-pQTLs and clinical diagnoses and laboratory measurements. Equally importantly, the summary statistics from their GWAS and PheWAS analyses have been made available to the broader scientific community at https://bidmc-cardiology-2024.shinyapps.io/pqtl_phewas_explorer/, allowing other researchers to benefit from the authors’ hard work in assembling and analyzing multiple databases. The scientific community must continue to recognize and value the efforts of researchers doing the work required to publish results of population science analyses in diverse populations.

## Version 1 11/01/2024

Electronic publication
